# Supplementary material for: Health-related quality of life implications of plantar ulcers resulting from neuropathic damage caused by leprosy: An analysis from the trial of autologous blood products (TABLE trial) in Nepal
Source: PLoS One. 2025 Feb 11;20(2):e0315944. doi: 10.1371/journal.pone.0315944 (PMC11813150; doi:10.1371/journal.pone.0315944)
Supplement: S1 Table — (DOCX) [file pone.0315944.s001.docx]

Supporting information S1

S1 Table: Comparing disutility model under different family and link assumptions

|  | Gaussian Family, identity link | | Gamma Family, identity link | | Gaussian Family, log link | |
| --- | --- | --- | --- | --- | --- | --- |
|  | Estimate | SE | Estimate | SE | Estimate | SE |
| Intercept | 0.35 | 0.23 | 0.35 | 0.24 | -0.96* | 0.48 |
| Ulcer | 0.11*** | 0.02 | 0.11*** | 0.02 | 0.27*** | 0.06 |
| Female (Male = reference) | 0.10* | 0.04 | 0.09* | 0.04 | 0.20** | 0.07 |
| Age | 0.00* | 0.00 | 0.00* | 0.00 | 0.00* | 0.00 |
| BMI | 0.01. | 0.00 | 0.01 | 0.00 | 0.01. | 0.01 |
| Systolic bp | 0.00 | 0.00 | 0.00* | 0.00 | -0.01 | 0.00 |
| Diastolic bp | 0.00 | 0.00 | 0.00 | 0.00 | 0.00 | 0.01 |
| Platelet result | 0.00 | 0.00 | 0.00 | 0.00 | 0.00 | 0.00 |
| Haemoglobin | 0.00 | 0.01 | 0.00 | 0.01 | -0.01 | 0.02 |
| Fasting blood sugar | 0.00 | 0.00 | 0.00 | 0.00 | 0.00 | 0.00 |
| Years since leprosy diagnosis | 0.00 | 0.00 | 0.00 | 0.00 | 0.00 | 0.00 |
| Antibiotics  Completed | -0.05 | 0.06 | -0.04 | 0.06 | -0.07 | 0.11 |
| Antibiotics  Unknown | -0.04 | 0.07 | -0.07 | 0.07 | 0.02 | 0.13 |
| Nerve enlarged in either leg | 0.00 | 0.03 | -0.01 | 0.03 | 0.01 | 0.07 |
| Loss of motor function in either foot | 0.02 | 0.03 | 0.02 | 0.03 | 0.05 | 0.05 |
| Deformity in foot | -0.03 | 0.04 | -0.02 | 0.04 | -0.10 | 0.08 |
| More than one ulcer | 0.06. | 0.04 | 0.07. | 0.04 | 0.13 | 0.08 |
| Weeks the trial ulcer has been unhealed | 0.00 | 0.00 | 0.00 | 0.00 | 0.00 | 0.00 |
| Trial ulcer is recurrent | 0.04 | 0.03 | 0.04 | 0.03 | 0.07 | 0.06 |
| Baseline ulcer area cm2 (measured using the PUSH tool | 0.00 | 0.00 | 0.00 | 0.00 | 0.01 | 0.01 |
|  | | | | | | |
| QIC | 109.8 |  | 513.4 |  | 514.3 |  |
| Significance codes: 0 ‘***’ 0.001 ‘**’ 0.01 ‘*’ 0.05 ‘.’ 0.1 ‘ ’ 1 | | | | | | |
