# Supplementary material for: Health-related quality of life implications of plantar ulcers resulting from neuropathic damage caused by leprosy: An analysis from the trial of autologous blood products (TABLE trial) in Nepal
Source: PLoS One. 2025 Feb 11;20(2):e0315944. doi: 10.1371/journal.pone.0315944 (PMC11813150; doi:10.1371/journal.pone.0315944)
Supplement: S2 Table — (DOCX) [file pone.0315944.s002.docx]

Supporting information S3

S3 Table: Correlation between residuals in linear model

|  | resid.1 | resid.5 | resid.9 | resid.13 | resid.17 | resid.21 | resid.32 |
| --- | --- | --- | --- | --- | --- | --- | --- |
| resid.1 | 1.00 | 0.43 | 0.10 | 0.14 | 0.30 | 0.25 | 0.29 |
| resid.5 | 0.43 | 1.00 | 0.41 | 0.39 | 0.46 | 0.17 | 0.25 |
| resid.9 | 0.10 | 0.41 | 1.00 | 0.52 | 0.61 | 0.41 | 0.11 |
| resid.13 | 0.14 | 0.39 | 0.52 | 1.00 | 0.65 | 0.78 | 0.18 |
| resid.17 | 0.30 | 0.46 | 0.61 | 0.65 | 1.00 | 0.59 | 0.07 |
| resid.21 | 0.25 | 0.17 | 0.41 | 0.78 | 0.59 | 1.00 | 0.38 |
| resid.32 | 0.29 | 0.25 | 0.11 | 0.18 | 0.07 | 0.38 | 1.00 |
