# Supplementary material for: Health-related quality of life implications of plantar ulcers resulting from neuropathic damage caused by leprosy: An analysis from the trial of autologous blood products (TABLE trial) in Nepal
Source: PLoS One. 2025 Feb 11;20(2):e0315944. doi: 10.1371/journal.pone.0315944 (PMC11813150; doi:10.1371/journal.pone.0315944)
Supplement: S1 Fig — (DOCX) [file pone.0315944.s003.docx]

Supporting information S2

S2 Figure: Comparing disutility models using QQ plots under different family and link assumptions

| Gaussian Family, Identity Link  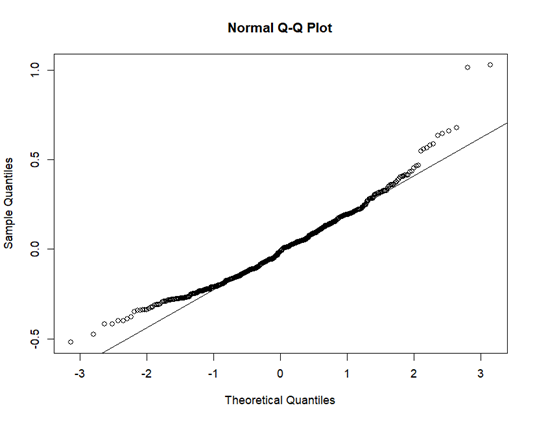 |
| --- |
| Gamma Family, Identity Link  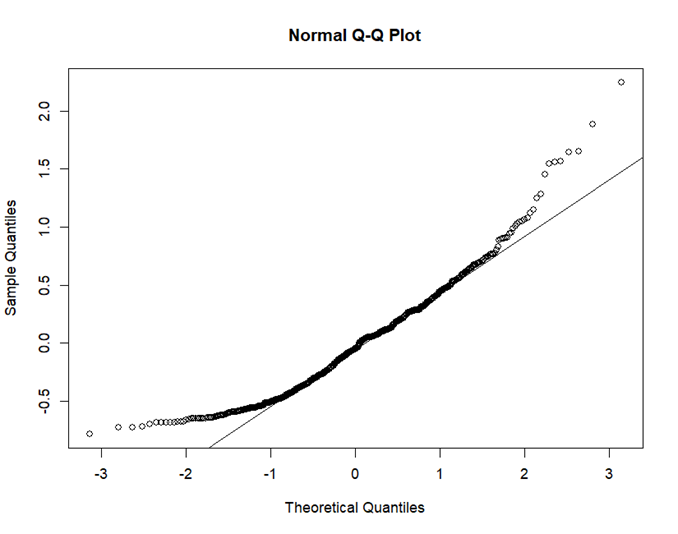 |
| Gaussian Family, Log Link  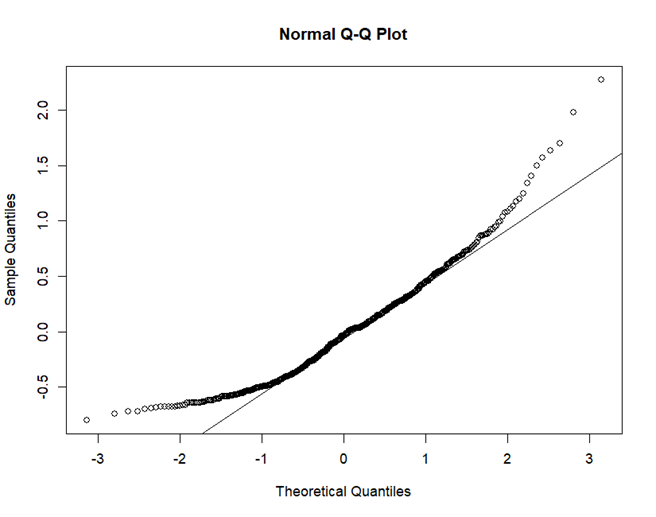 |
