# Supplementary material for: Health-related quality of life implications of plantar ulcers resulting from neuropathic damage caused by leprosy: An analysis from the trial of autologous blood products (TABLE trial) in Nepal
Source: PLoS One. 2025 Feb 11;20(2):e0315944. doi: 10.1371/journal.pone.0315944 (PMC11813150; doi:10.1371/journal.pone.0315944)
Supplement: Attachment — Submitted filename: Response to reviewers.docx [file pone.0315944.s004.docx]

We thank reviewer one for their considered review of our paper and for highlighting areas for improvement. Below we include our response and related changes.

| **Reviewer comment** | **Response** | **Changes** |
| --- | --- | --- |
| **Introduction** | | |
| line 54 - It would be more appropriate to use 'mycobacterial' instead of 'bacterial' in this context, as it provides greater specificity and accurately reflects the genus of the pathogen involved in leprosy. | Thank you for providing this information. We have made the change. | Line 54 “bacterial” changed to “mycobacterial” |
| line 62 - Further clarification regarding the rationale behind selecting plantar ulcers as the primary burden, given that neuropathic damage in leprosy can present with numerous clinical manifestations (For example chronic neuropathic pain in leprosy treated patients) would provide more background to the reader.  Additionally, it would be valuable to provide more regional epidemiological data on the prevalence and impact of plantar ulcers in Nepal to better contextualize this choice. This would enhance the understanding of the specific burden of plantar ulcers in comparison to other neuropathic complications associated with leprosy. | We agree that providing some more context and epidemiological data will improve the introduction and have added sentences to this effect. We hope this, combined with the references to the clinical paper and trial protocol, provide the clarification needed. Our paper is aiming to estimate the HRQoL decrement associated with ulcers caused by leprosy related neuropathic damage given that this trial has already taken place and therefore the HRQoL data is available rather than justifying the original trial. | Sentences added to the introduction. Line 62.  *“Nepal is one of the World Health Organisation’s (WHO) global priority countries for their Global Leprosy Strategy 2021 - 2030 due to prevalence and new case detection, estimated at 3249 in 2018 (5, 6). Beliefs regarding leprosy resulting from former life transgressions and associated stigma in Nepal can prevent patients seeking treatment resulting in ulcers and the requirement of inpatient hospital care (7). Plantar ulcers are those which occur on the underside of the foot. Plantar ulcer healing requires reducing the pressure on the ulcer but due to the physical work requirements of many of the patients this is impractical creating a cycle between poverty and ill health.”* |
| **Methods** | | |
| line 103 - Although it is noted that details are provided in a previous publication, given the significance of the topic, it would be important to clarify whether comorbidities that can also cause neuropathic damage, such as diabetes, physical injury (trauma), infections, autoimmune diseases, toxins, chemotherapy, medications, and other causes were excluded. | We agree that it would be useful to provide this information here rather than only referring to the previous publication. | Sentence added to the first paragraph in the Methods section under the subheading “TABLE trial”. Lines 113 – 115.  *“Patients were excluded from the trial if they had significant medical conditions such as diabetes, HIV, chronic HEP B, chronic HEP C or TB, full exclusion criteria are provided in the trial results (10).”* |
| line 119 - During the EQ-5D questionnaire application, respondents were instructed to base their responses specifically on the impact of the plantar ulcer alone? Do the reported health-related quality of life outcomes accurately reflect the burden of the ulcer itself? Or other concurrent health conditions could influence their responses. (Please add any statement if needed in the limitations sections of the manuscript) | The EQ5D is designed to capture the patients’ overall health and not only relate to the plantar ulcer. We have added a sentence to the first paragraph, under the subtitle utility data, to clarify this. It is the aim of the regression analysis to isolate the effect of the plantar ulcer on HRQoL, we do not ask the patients to do this in their responses to the questionnaire. | First paragraph under utility data. Line 132.  *“This questionnaire is designed to capture patients’ overall level of health, not just the impact of a specific illness, in this case the ulcer.”* |
| **Results** | | |
| Table 1 - The "more than one ulcer" variable would reflect ulcers in the same location (plantar or different parts of the body?) | We agree that we should add this detail. It refers to more than one plantar ulcer. We have updated the text accordingly. | Table 1: *“More than one ulcer” changed to “More than one plantar ulcer”* |
| Table 4 - It would be beneficial to include a clear explanation of what the intercept (first line of the table) represents in the math modelling, articulated in simple terms. This would enhance the accessibility of the results and ensure that readers without a strong statistical background can fully understand its significance in the context of the analysis. | We agree this information will enhance accessibility of the results to more readers. | We have added the following sentence in the paragraph immediately before table 4. Lines 225 to 233.  “*The results provide estimates of the disutility whilst controlling for covariates (Table 4). The intercept can be interpreted as the disutility if all covariates are set to zero; however, as many of the covariates cannot be zero in this context (i.e. age, years since leprosy diagnosis) it is not perfectly interpretable. Despite this, since the coefficients are very small, except for ulcer and female, we can broadly interpret the constant as the disutility for males with no ulcer, in this patient group. This implies a HRQoL score of 0.65 (a score of 1 = full health) which demonstrates these patients are experiencing health problems even without accounting for the ulcer.”* |
| **Discussion** | | |
| Is there any consideration of potential cultural factors that might influence respondents to report improvements in their condition as a form of respect or gratitude for receiving assistance? It would be useful to reference any published studies that have explored these social dynamics, particularly in similar healthcare contexts, as this could impact the accuracy of self-reported outcomes. | This is a very valid point. There are many risks of bias with self-reported outcome measures, and these may vary depending on the cultural context. We have added text and references to this effect in the discussion section. | The last sentence of the 7^th^ paragraph in the discussion. Lines 319 to 323.  *“Self-reported health-related quality of life measures are subject to many forms of bias, which may also vary depending on the cultural context. One form of bias is collection mode related bias in which patients may provide responses according to what they believe researchers would like to collect, it is possible this may be more prevalent in countries with a certain cultural norms (24, 25).”* |

We would also like to draw the editors attention to the amended Role of Funder statement which is included in the manuscript and stated here:

This research was funded by the National Institute for Health Research (NIHR) (NIHR200132) using UK aid from the UK Government to support global health research. RJL is also funded by NIHR Applied Research Collaboration (ARC) West Midlands and Midlands Patient Safety Research Collaboration (PSRC). The views expressed in this publication are those of the author(s) and not necessarily those of the NIHR or the UK Department of Health and Social Care. The funders had no role in study design, data collection and analysis, decision to publish, or preparation of the manuscript.
